# Effects of Low-Level Laser Therapy on M1-Related Cytokine Expression in Monocytes via Histone Modification

**DOI:** 10.1155/2014/625048

**Published:** 2014-02-19

**Authors:** Chia-Hsin Chen, Chau-Zen Wang, Yan-Hsiung Wang, Wei-Ting Liao, Yi-Jen Chen, Chang-Hung Kuo, Hsuan-Fu Kuo, Chih-Hsing Hung

**Affiliations:** ^1^Department of Physical Medicine and Rehabilitation, Faculty of Medicine, College of Medicine, Kaohsiung Medical University, Kaohsiung, Taiwan; ^2^Department of Physical Medicine and Rehabilitation, Kaohsiung Medical University Hospital, Kaohsiung, Taiwan; ^3^Department of Physical Medicine and Rehabilitation, Kaohsiung Municipal Ta-Tung Hospital, Kaohsiung, Taiwan; ^4^Orthopaedic Research Center, Kaohsiung Medical University, Kaohsiung, Taiwan; ^5^Department of Physiology, Kaohsiung Medical University, Kaohsiung, Taiwan; ^6^School of Dentistry, College of Dental Medicine, Kaohsiung Medical University, Kaohsiung, Taiwan; ^7^Department of Biotechnology, Kaohsiung Medical University, Kaohsiung, Taiwan; ^8^Department of Pediatrics, Kaohsiung Medical University Hospital, Kaohsiung Medical University, Kaohsiung, Taiwan; ^9^Division of Cardiology, Department of Internal Medicine, Kaohsiung Medical University Hospital, Kaohsiung, Taiwan; ^10^Division of Cardiology, Department of Internal Medicine, Kaohsiung Municipal Ta-Tung Hospital, Kaohsiung, Taiwan; ^11^Department of Pediatrics, Faculty of Pediatrics, College of Medicine, Kaohsiung Medical University, Kaohsiung, Taiwan; ^12^Department of Pediatrics, Kaohsiung Municipal Hsiao-Kang Hospital, Kaohsiung Medical University Hospital, Kaohsiung Medical University, No. 482, Shanming Road, Siaogang District, Kaohsiung 80708, Taiwan

## Abstract

Low-level laser therapy (LLLT) has been used in the treatment of radiotherapy-induced oral mucositis and allergic rhinitis. However, the effects of LLLT on human monocyte polarization into M1 macrophages are unknown. To evaluate the effects of LLLT on M1-related cytokine and chemokine production and elucidate the mechanism, the human monocyte cell line THP-1 was treated with different doses of LLLT. The expression of M1-related cytokines and chemokines (CCL2, CXCL10, and TNF-**α**) was determined by ELISA and real-time PCR. LLLT-associated histone modifications were examined by chromatin immunoprecipitation (ChIP) assays. Mitochondrial involvement in the LLLT-induced M1-related cytokine expression was evaluated by quantitative real-time PCR. Flow cytometry was used to detect the cell surface markers for monocyte polarization. The results showed that LLLT (660 nm) significantly enhanced M1-related cytokine and chemokine expression in mRNA and protein levels. Mitochondrial copy number and mRNA levels of complex I-V protein were increased by LLLT (1 J/cm^2^). Activation of M1 polarization was concomitant with histone modification at TNF-**α** gene locus and *IP-10* gene promoter area. This study indicates that LLLT (660 nm) enhanced M1-related cytokine and chemokine expression via mitochondrial biogenesis and histone modification, which may be a potent immune-enhancing agent for the treatment of allergic diseases.

## 1. Introduction

Both innate and adaptive immune responses are, in every way, affected by polarization with cytokines. The expression of costimulatory molecules and chemokines, as well as the execution of effector programs, is affected in monocytes. In humans and mice, T helper (Th)1 and Th2 polarization with IFN-r and IL-4 is well understood [1, 2]. Polarized macrophages are also generally classified into two groups: M1 and M2 macrophages. IL-4 polarization, also known as either alternative or M2a activation, stimulates wound recovery and parasite immunity responses. IFN-r polarization, which is referred to as either classical or M1 activation, is responsible for tumor resistance, intracellular killing, and IL-12 production in monocytes [3]. M1 macrophages, which are activated by the classical pathway, are shown to be responsive to two signals: type 1 inflammatory cytokines and microbial products [4].

There are three subsets of M2 macrophages: M2a, induced by IL-4 or IL-13; M2b, induced by immune complexes and agonists of TLRs or IL-1 receptors; and M2c, induced by IL-10 and glucocorticoid hormones [3]. M1 and M2 macrophages can be differentiated based on their receptors, expression of cytokines and chemokines, and effector function. M1 macrophages are microbicidal and inflammatory, and M2 macrophages are immunomodulators (M2a and M2c) and possess minimal microbicidal effects. Consequently, the activation of macrophages is either proinflammatory or anti-inflammatory. Recently, the activation or polarization of macrophages has been demonstrated to be rapid, plastic, and fully reversible. This shows that macrophages are dynamic when they first engage in the inflammatory response and the resolution process that follows [5] and that changes in function are caused by changes in the microenvironment [6].

Low-level laser therapy (LLLT) is a form of light emission with a power output of less than 500 mW and is therefore considered nonthermal irradiation to living tissue. LLLT has been proposed to have various therapeutic effects. LLLT is known to be a noninvasive treatment modality and has been applied in various fields. LLLT was thought to be effective in pain relief and promoting recovery of some pathology, including tendinopathies, osteoarthritis, temporomandibular joint disorders, wound healing, and nerve injuries [7, 8]. The exact mechanism is still under investigation, but the mechanism is likely to be photochemically related. This would affect the biological regulation of nitric oxide and adenosine triphosphate and would further affect the inflammatory process or cytokine release. LLLT is prevalent in the prevention and treatment of cancer therapy-induced oral mucositis [9, 10] and may alter human immunity. LLLT has also been shown to have several biological effects that favor the healing process [11]. LLLT (660 nm) is able to promote the skin repair of burned rats by decreasing the necrotic area and upregulating cyclooxygenase-2 and vascular endothelial growth factor expression [12]. An in vitro study demonstrated that increased intracellular calcium influx occurred in mast cells, followed by histamine release after laser irradiation, which may explain the biological effect of LLLT in promoting wound healing [13]. Cytokine expression in short-term muscle remodeling is also modulated by LLLT, which leads to a decline in TNF-*α* and TGF-*β* after cryoinjury [14]. Similarly, the clinical value of the potential immune modulation effect of laser therapy has recently been studied in the treatment of allergic rhinitis. The ability of the KTP/532 YAG laser to reduce nasal congestion and discharge in patients with allergic rhinitis has been identified. The KTP/532 YAG laser is effective as an additional treatment for patients who are refractory to medications, and the treatment is extremely well tolerated without significant side effects. After one year, nasal obstruction was improved in 69% of cases and nasal discharge in 40% of cases [15]. 308 nm xenon chloride (XeCl) UVB irradiation significantly minimized these symptoms, including rhinorrhoea, sneezing, and nasal obstruction, and improved the total nasal scores and the allergen-induced skin prick tests in a dose-dependent manner. The XeCl UVB excimer laser may also serve as a new treatment option for treating allergic rhinitis [16], which is a Th2-dominant disease that is suppressed by Th1 or M1 immunity.

Controlled by the action of histone acetyltransferases (HATs), histone deacetylases (HDACs), and methyltransferases, histone acetylation and methylation are important epigenetic modifications that influence gene transcription [17]. Chromatin carries numerous histones and DNA modifications that are associated with gene transcription [17]. Modifications on histones, such as acetylation or trimethylation at H3K4, H3K36, and H3K79, are associated with gene activation [17]. It is unknown, however, whether LLLT modulates human monocyte polarization and immune function via epigenetic regulation. Because different types of lasers have been used for the treatment of Th2-dominant disease, we evaluate the influence of LLLT on monocyte polarization in this study. We investigated the regulatory effects of LLLT on monocyte M1 polarization to provide evidence for the use of LLLT for immunologic disorders.

## 2. Materials and Methods

### 2.1. Cell Preparation

The human monocytic cell line THP-1 (American Type Culture Collection, Rockville, MD) was cultured in RPMI 1640 medium (Sigma-Aldrich, St. Louis, MO) supplemented with 10% fetal bovine serum, 100 U/mL of penicillin, and 100 *μ*g/mL of streptomycin at 37°C with 5% CO_2_ in a humidified incubator. THP-1 cells were centrifuged, resuspended in fresh media, and plated in 24-well plates at a cell density of 5 × 10^5^/mL 24 hours before experimental use. The cells were pretreated with a low-power gallium-aluminum-arsenide (GaA1As) laser (0–3 J/cm^2^; 660 or 808 nm) alone or 2 hours before LPS (0.2 µg/ml) stimulation. Cell supernatants were collected 12, 24, and 48 hours after LPS stimulation. To investigate epigenetic regulation, the cells were pretreated with methylthioadenosine (MTA, a histone methyltransferase inhibitor) or anacardic acid (AA, a histone acetyltransferase inhibitor) 1 hour before LLLT. To investigate the mitochondria involvement in LLLT-related monocyte polarization, the cells were pretreated with oligomycin (1 and 2.5 *μ*g/mL, Sigma-Aldrich, St. Louis, MO, USA) or antimycin (0.1 and 0.5 *μ*g/mL, Sigma-Aldrich, St. Louis, MO, USA) 1 hour before LLLT [18].

### 2.2. Low-Level Laser Irradiation Treatment

The GaA1As ultra red laser with wavelengths of 660 nm and GaA1As near-infrared laser with wavelengths of 808 nm (Transverse Ind. Co., Ltd., Taipei, Taiwan) were used as the laser source in our study. The laser characteristics for laser description were shown in Table 1. A total volume of 1 mL of cell-containing media for 12-well plates was added into each well to decrease the refraction during the low-level laser irradiation treatment. The distance between the GaA1As laser source and the culture plate was adjusted to ensure homogeneous laser exposure in 12-well plates. The cells were treated with the GaA1As laser beam to reach a total energy of 0, 1, 2, and 3 J/cm^2^, respectively. Cell supernatants were collected 12, 24, and 48 hours after irradiation.

### 2.3. RNA Extraction and Real-Time PCR

THP-1 cells were treated with different doses of LLLT and total RNA was isolated from cells immediately (*t* = 0) or 6 hours after LPS stimulation. Total RNA was extracted from cells using TRIzol (Invitrogen, Carlsbad, CA) according to the manufacturer's instruction. Three *μ*g of RNA from each sample was then reverse-transcribed into first-strand cDNA in 20 *μ*L of reaction mixture using the SuperScript First-Strand Synthesis System with the Real-time PCR kit (Invitrogen). Measurements were performed by an ABI PRISM 9700 HT sequence detection system (Applied Biosystems, Foster City, CA) using a predeveloped Taqman probe/primer combination for M1-related genes and glyceraldehyde 3-phosphate dehydrogenase (G3PDH) from the same cDNA samples. Taqman PCR was performed in 10 *μ*L using AmpliTaq Gold polymerase and the universal master mix (Applied Biosystems). Threshold cycle numbers were transformed using the comparative threshold cycle and relative value methods according to the manufacturer's recommendation and expressed relative to G3PDH, which is used as a housekeeping gene by multiplexing single reactions. The M1-related cytokine and chemokine genes are as follows: CCL2/MCP-1, CXCL10/IP-10, and TNF-*α*.

### 2.4. ELISA Assay

The CCL2/MCP-1, CXCL10/IP-10 and TNF-*α* concentrations in the cell supernatants were determined using commercially available ELISA-based assay systems (R&D Systems, Minneapolis, MN). Assays were performed using the protocols recommended by the manufacturer.

### 2.5. Chromatin Immunoprecipitation Assay (ChIP)

5 × 10^5^ cells were treated with 1% formaldehyde for 10 min at room temperature, followed by sonication of the DNA and immunoprecipitation of chromatin overnight with antibodies against acetylated H3 and H4 and trimethylated H3K4 (Upstate Biotechnology, Waltham, MA). Immune complexes were collected using a protein A slurry (Invitrogen), and the DNA was reverse cross-linked, extracted, and quantified using a Taqman SDS 7900HT. For PCR amplification of ChIP products, primers and probes were designed to analyze the proximal promoter and intronic enhancer regions of the TNF-*α* gene as previously described [19, 20], encompassing the following subregions relative to the transcription start site: TNF1 (T1, +99 to –42); TNF2 (T2, +32 to −119); TNF3 (T3, –100 to –250); TNF4 (T4, –195 to –345); and +1417, +720, and −1700. PCRs were performed using the ABI 7700 Taqman thermocycler. Primers and probes were also designed to analyze the proximal promoter regions of the CXCL10/IP-10 gene (CXCL10/IP-10-1: +9 to −172 and CXCL10/IP-10-2: −444 to −622) [21]. PCRs were run on the ABI 7700 Taqman thermocycler. All Taqman reagents were purchased from Applied Biosystems. The relative intensities of the amplified products were normalized to the input DNA.

### 2.6. Flow Cytometry Analysis

THP-1 cells were cultured at 10^6^/mL in 12-well round-bottom plates (1 mL/well), treated with LLLT (660 nm), and incubated for 24 h. The cells were harvested and washed 3 times with PBS for direct immunofluorescence staining using labeled monoclonal antibodies to CD14, CD45RO, CCR7, or CD86. All fluorescence- conjugated monoclonal antibodies were purchased from eBioscience. The cell surface markers were analyzed using a FACScan flow cytometer and the CellQuest software (Becton Dickinson, Franklin Lakes, NJ, USA).

### 2.7. Quantitative Real-Time PCR (Q-PCR) for Mitochondrial Biogenesis

According to the manufacturer (Invitrogen, Carlsbad, CA), an anchored oligo-dT primer was used to reverse-transcribe total RNA (1 *μ*g) using SuperScript II. The DNA product then served as template for Q-PCR. Primer pairs were designed using Primer3 (http://frodo.wi.mit.edu/primer3/) and were validated using in silico PCR (http://genome.ucsc.edu/cgi-bin/hgPcr) and BLAST (http://blast.ncbi.nlm.nih.gov/Blast.cgi). The following primer sequences were used: MT-ND1—NADH dehydrogenase, subunit 1 (MT complex I) FW: ACCATTTGCAGACGCCATAA and RE: TGAAATTGTTTGGGCTACGG; SDHA—succinate dehydrogenase complex, subunit A, flavoprotein (MT complex II) FW: CAAACAGGAACCCGAGGTTTT and RE: CAGCTTGGTAACACATGCTGTAT; MT-CYTB—mitochondrial cytochrome b (MT complex III) FW: GCCCTCGGCTTACTTCTCTT and RE: GACGGATCGGAGAATTGTGT; COX1 (MT-COI)—cytochrome c oxidase I (MT complex IV) FW: TTCGCCGACCGTTGACTATTCTCT and RE: AAGATTATTACAAATGCATGGGC; MT-ATP6—ATP synthase, H+ transporting, mitochondrial Fo complex, subunit F6 (MT complex V) FW: TTTGCGGAGGAACATTGGTGT and RE: TCCAGATGTCTGTCGCTTAGAT; UCP2—uncoupling protein 2 (mitochondrial, proton carrier) FW: CCTGAAAGCCAACCTCATGAC and RE: CAATGACGGTGGTGCAGAAG; and 18 S rRNA FW: TAGAGGGACAAGTGGCGTTC and RE: CGCTGAGCCAGTCAGTGT.

For Q-PCR time course samples (*n* = 3), 10 *μ*L reactions consisting of 3 *μ*L of diluted cDNA and 0.3 *μ*M of forward and reverse gene-specific primers combined with 2× Power SYBR Green PCR Master Mix (Applied Biosystems, Foster City, CA) were aliquoted into 96-well plates using a Biomeck 2000 Laboratory Automation Workstation (Beckman Coulter Inc., Fullerton, CA). Applied Biosystems PRISM 7900HT Sequence Detection System was used for the amplification process that included a ten-minute 95°C denaturation stage, then forty repetitions of 95°C for fifteen seconds, and lastly 60°C for one minute. Quantifications were obtained by the comparative CT method (ΔΔCT) (Applied Biosystems, Foster City, CA). The geometric mean of housekeeping gene (GAPDH) expression served as the internal control. The relative copy number of mtDNA was computed via normalizing the crossing points in the quantitative PCR curves between the mitochondrial ND1 gene and the nuclear 18S rRNA gene, and the ratio was normalized to the control [22].

### 2.8. Statistical Analysis

All data are presented as the means ± SD. Differences between experimental and control groups were analyzed by using the Mann-Whitney *U*  test. Changes in chemokines and cytokines at different doses of LLLT alone were analyzed using the Wilcoxon signed rank test. A *P*-value < 0.05 was considered indicative of a significant difference between groups.

## 3. Results

### 3.1. M1-Related Chemokine and Cytokine mRNA Expression Was Modulated by LLLT in THP-1 Cells

**Figure 1:**
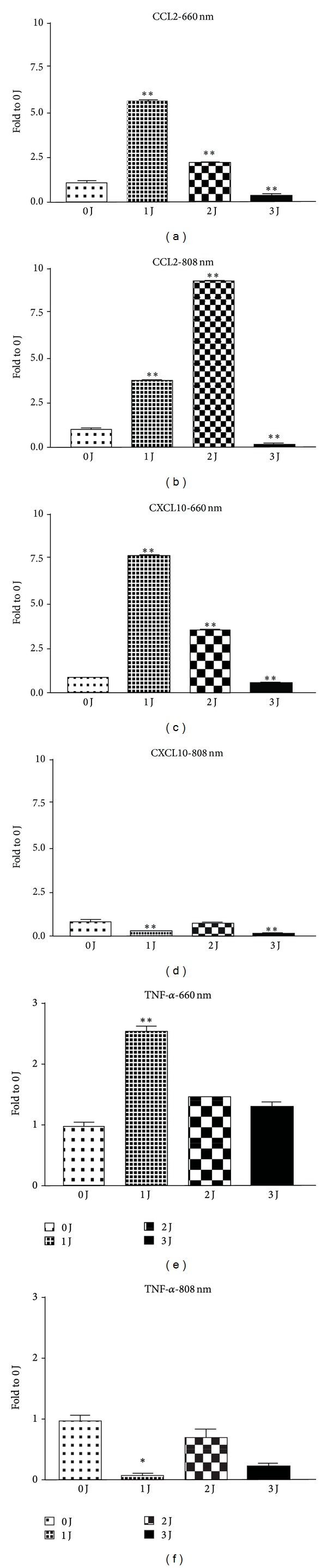
Effect of LLLT on M1-related chemokine and cytokine mRNA expression in THP-1 cells using real-time PCR analysis. M1-related chemokines CCL2 and CXCL10 expression was enhanced by 660 nm (1-2 J/cm^2^) and 808 nm (2 J/cm^2^) LLLT ((a) and (b)). However, 3 J/cm^2^ of LLLT (660 and 808 nm) suppressed CCL-2 expression in THP-1 cells. CXCL-10 mRNA expression was enhanced by 660 nm LLLT but was suppressed by 808 nm LLLT ((c) and (d)). TNF-*α* was also enhanced by 660 nm LLLT but was suppressed by 808 nm LLLT 24 hours after irradiation ((e) and (f)). *P < 0.05; **P < 0.01; and ***P < 0.001 between control and LLLT treatment.

**Figure 2:**
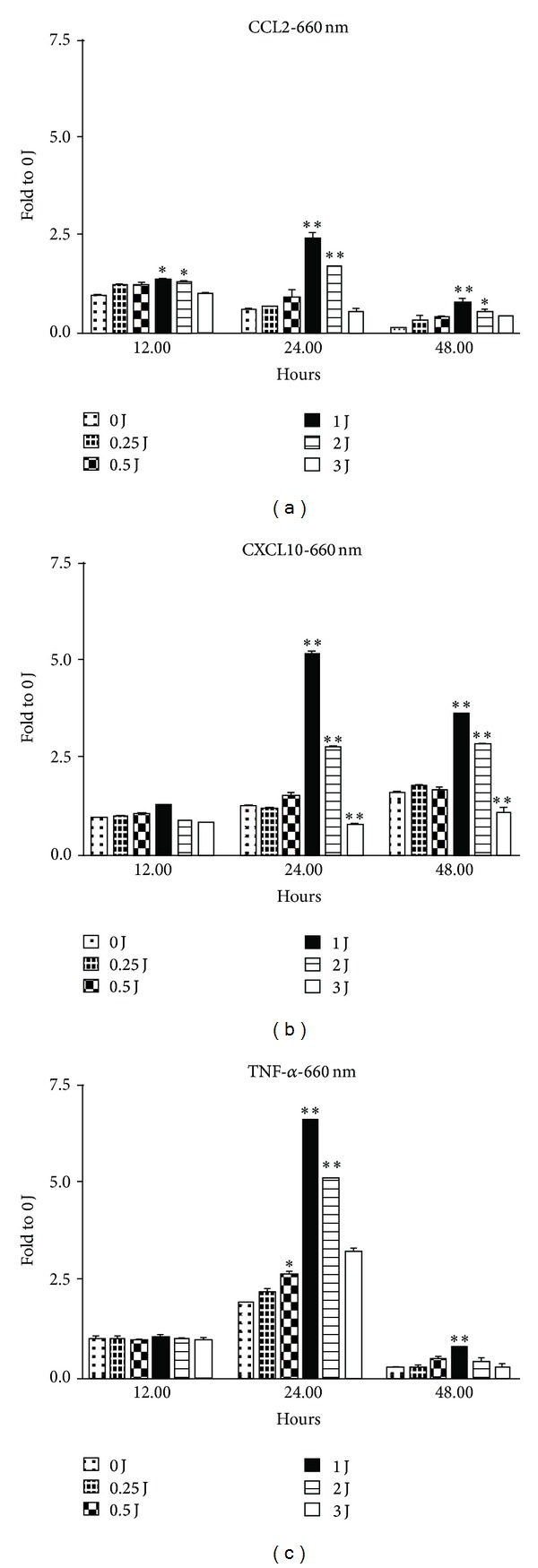
The effects of LLLT on the expression of M1-related cytokines and chemokines CCL2 (a), CXCL10 (b) and TNF-*α* (c) mRNA at 12 h, 24 h, and 48 h time points using real-time PCR analysis. *P < 0.05; **P < 0.01; and ***P < 0.001 between control and LLLT treatment.

We first tested whether LLLT (0–3 J/cm^2^, 660 and 808 nm) influenced the M1-related chemokine and cytokine expression in THP-1 cells. Real-time PCR data showed that the M1-related chemokine CCL-2 was enhanced by 660 nm (1-2 J/cm^2^) and 808 nm (1-2 J/cm^2^) LLLT 24 hours after irradiation. The most powerful effect was produced by 1 J/cm^2^ of 660 nm LLLT and 2 J/cm^2^ of 808 nm LLLT (Figures 1(a) and 1(b)). However, 3 J/cm^2^ of LLLT (660 and 808 nm) suppressed CCL-2 expression in THP-1 cells. CXCL-10 mRNA expression was enhanced by 660 nm LLLT but suppressed by 808 nm LLLT (Figures 1(c) and 1(d)). TNF-*α*, an M1-related pro-inflammatory cytokine, was also enhanced by 660 nm LLLT but suppressed by 808 nm LLLT 24 hours after irradiation (Figures 1(e) and 1(f)). The effect of LLLT on M1-related cytokine and chemokine mRNA expression was observed at 12, 24, and 48 h time points (Figures 2(a), 2(b), and 2(c)). There were no differences between the control group and all of the other groups with different doses of LLLT treatment, indicating that cell viability was not affected by LLLT (data not shown).

### 3.2. M1-Related Chemokine and Cytokine Protein Expression Was Modulated by LLLT in THP-1 Cells

**Figure 3:**
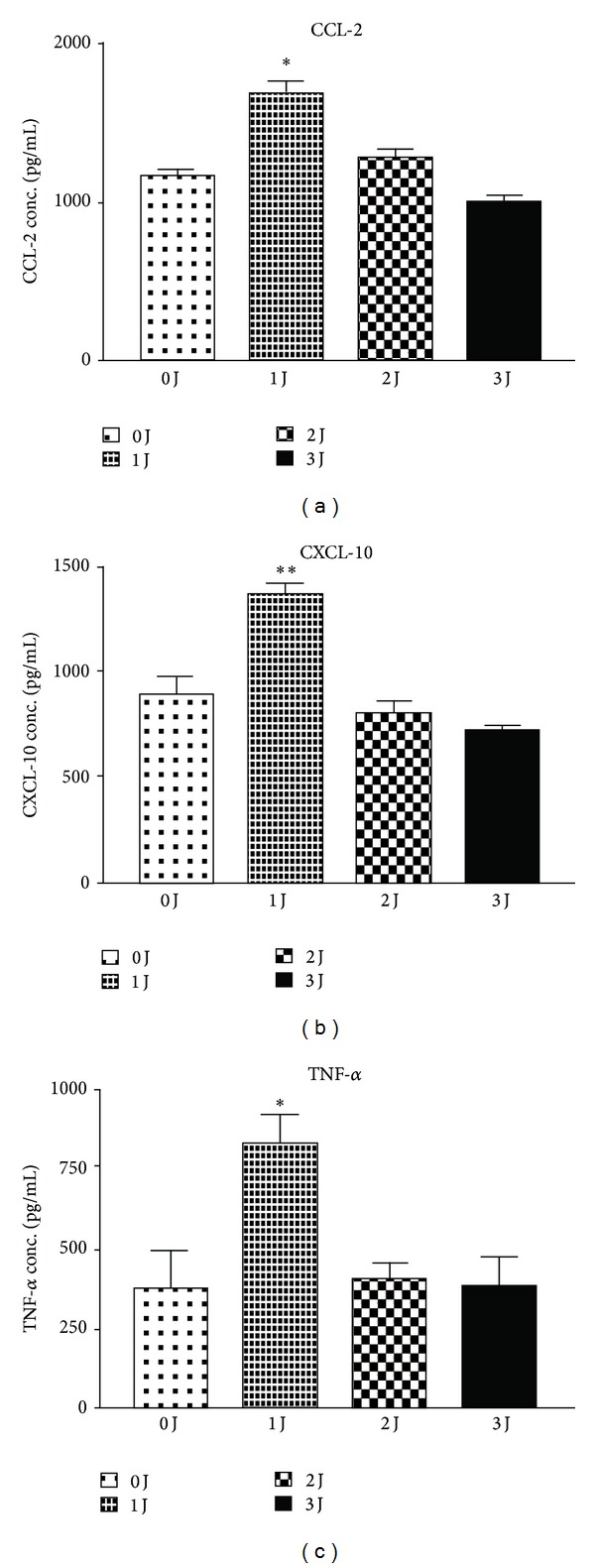
LLLT-induced M1-related CCL2 and CXCL10 protein expression in monocytes using ELISA assay. one J/cm^2^ of 660 nm LLLT significantly induced CCL2 (a), CXCL10 (b), and TNF-*α* (c) production in THP-1 cells, whereas either 2 J/cm^2^ or 3 J/cm^2^ did not. *P < 0.05; **P < 0.01; and ***P < 0.001 between control and LLLT treatment.

Because LLLT could induce M1-related cytokine and chemokine mRNA expression in monocytes, we examined whether LLLT could also induce M1-related cytokine and chemokine protein expression. one J/cm^2^ of 660 nm LLLT significantly induced CCL2 and CXCL10 production in human monocytes, whereas 2 J/cm^2^ and 3 J/cm^2^ did not (Figures 3(a) and 3(b)). TNF-*α* protein production was also enhanced by 660 nm LLLT 24 h after irradiation (Figure 3(c)).

**Figure 4:**
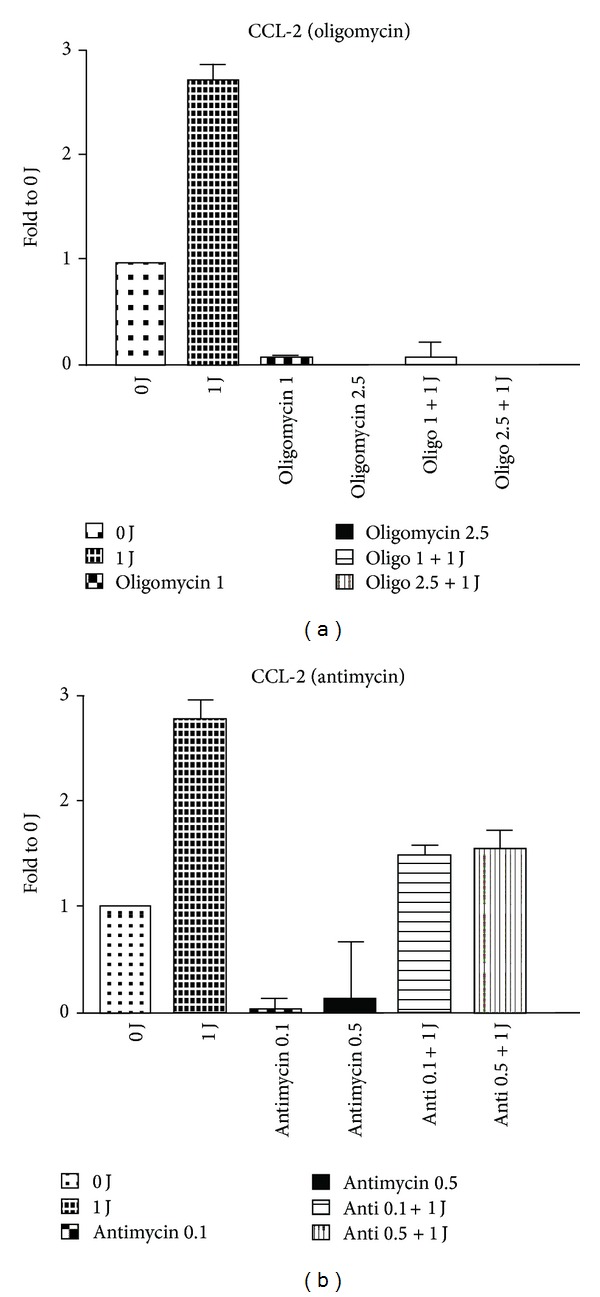
Real-time PCR results showed that 1 J/cm^2^ of 660 nm LLLT-induced CCL2 mRNA expression was inhibited by oligomycin (a) and antimycin (b). *P < 0.05 between LPS and LPS plus LLLT treatment.

**Figure 5:**
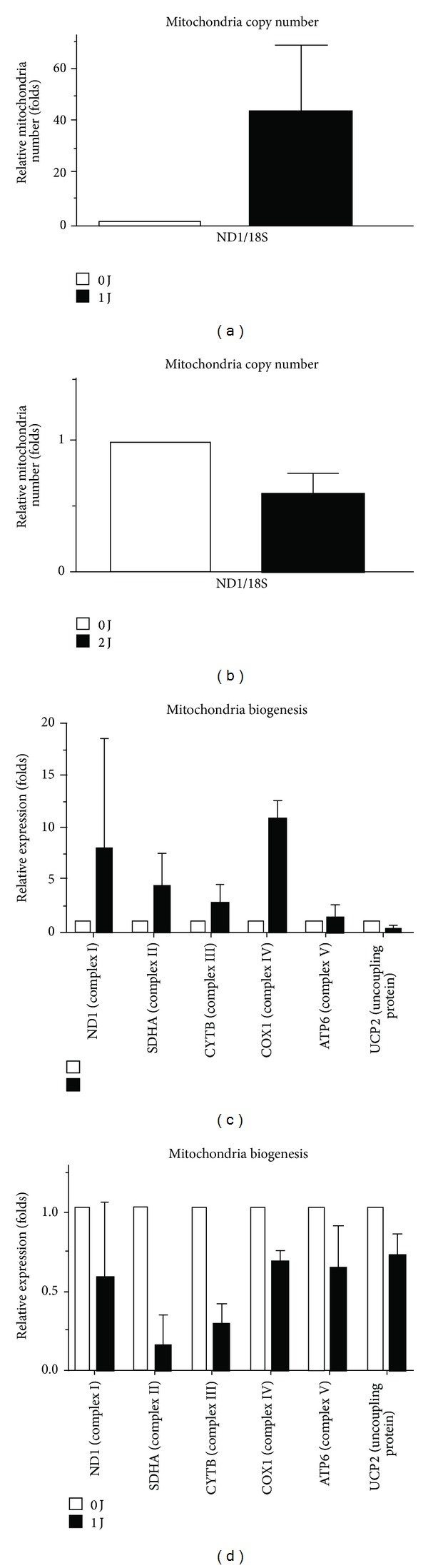
One J/cm^2^ of LLLT increased the copy number of mitochondria, whereas 2 J/cm^2^ did not ((a) and (b)). One J/cm^2^ of LLLT increased the mRNA expression of complexes I to V and uncoupling protein, whereas 2 J/cm^2^ did not ((c) and (d)).

The influence of LLLT on M1-related chemokine and cytokine production may also involve mitochondrial biogenesis and activation. It is known that oligomycin hinders ATP synthase by blocking its proton channel (Fo subunit), which is necessary for oxidative phosphorylation of adenosine diphosphate to ATP and leads to an increased proton gradient, which decreases both respiratory activity and oxidative phosphorylation, thereby resulting in mitochondrial dysfunction [23]. Antimycin is a mitochondrial inhibitor that binds in the energy-coupling site and inhibits the flow of electrons from cytochrome b to cytochrome c_1_. Low-intensity laser irradiation has been reported to improve mitochondrial dysfunction and leads to mitochondrial alterations [24, 25]. The inhibition of LLLT-induced CCL2 mRNA expression by oligomycin and antimycin suggested mitochondrial involvement (Figure 4). Therefore, we measured mitochondrial copy number after LLLT. As shown in Figures 5(a) and 5(b), 1 J/cm^2^ of LLLT significantly increased the copy number of mitochondria, but 2 J/cm^2^ of LLLT did not. The data are similar to the LLLT-induced production of M1-related chemokine and cytokine. Next, we evaluated the involvement of respiratory chains including complexes I to V and uncoupling protein. one J/cm^2^ of LLLT increased the mRNA amount of complexes I to V and uncoupling protein, whereas 2 J/cm^2^ did not (Figures 5(c) and 5(d)).

### 3.3. LLLT-Mediated Histone Modifications at the TNF-*α* Gene Locus

**Figure 6:**
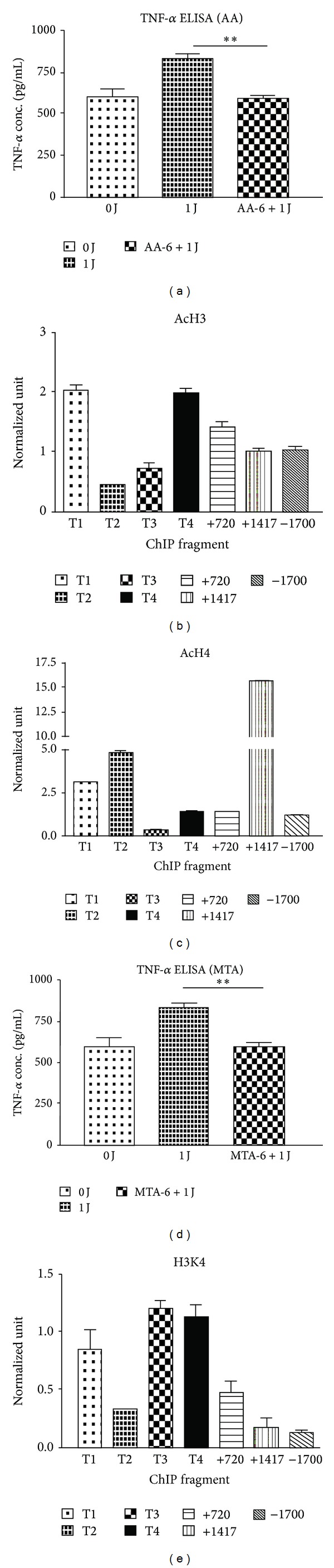
The involvement of histone acetylation and methylation in the regulatory effect of LLLT on TNF-*α* expression. The levels of TNF-*α* expression in LLLT-treated THP-1 cells in the presence or absence of AA (a) and MTA (d) using ELISA assay. ChIP analysis of the relative levels of acetylated H3 (AcH3) (b), acetylated H4 (AcH4) (c), and trimethylated H3K4 (e) at the TNF-*α* gene locus. ChIP encompassed the following subregions relative to the transcription start site: TNF1 (T1, +99 to –42), TNF2 (T2, +32 to –119), TNF3 (T3, –100 to –250), TNF4 (T4, –195 to –345), +1417, +720, and –1700. The relative levels were normalized to the input DNA and shown as the means (±SD) of 3 individual study subjects. **P < 0.01 was considered significant compared with the control without inhibitor.

Epigenetic regulation is one of the important control mechanisms for TNF-*α* expression. It has been shown that epigenetic modification at the TNF-*α* gene locus occurs by a coordinated and complicated network of regulation involving DNA methylation, histone modification, and chromatin remodeling. Studies in monocytes and macrophages have shown that although there are different patterns of histone modifications, the main regulatory regions associated with histone modifications could be identified in LLLT-treated monocytes [19]. In fact, the involvement of histone acetylation in the regulation of TNF-*α* expression was further supported by the finding that AA significantly suppressed TNF-*α* expression in LLLT-treated THP-1 cells (Figure 6(a)). To determine whether histone modifications occurred in theTNF-*α* gene locus in monocytes, ChIP analysis of THP-1 cells treated with LLLT was conducted; PCR primers corresponding to four overlapping subregions (−1700 and TNF1–4, covering the region between −345 and +99) in the TNF-*α* promoter and two intronic regions (+720 and +1417) in the TNF-*α* gene were used. Compared to the histone modifications found in the medium control cultures, significant histone modifications were detected at the TNF-*α* gene locus in LLLT-treated THP-1 cells. As shown in Figures 6(b) and 6(c), upregulated TNF-*α* expression in LLLT-treated THP-1 cells was associated with an increased level of histone 3 acetylation primarily in the T1, T4, and intron sequence (+1720) of the TNF-*α* gene; however, increased histone 4 acetylation was found to be mostly associated with the proximal promoter regions of the TNF-*α* gene in the T1, T2, and intron sequence (+1417).

Epigenetic regulation by histone methylations can be another mechanism for gene expression control [17]. We next examined whether the effects of LLLT on TNF-*α* expression were due to histone methylation. THP-1 cells were pretreated with MTA before LLLT treatment. LLLT-enhanced TNF-*α* expression was reversed by MTA (Figure 6(d)). Moreover, ChIP analysis also showed elevated levels of tri-methylated H3K4 at the proximal promoter subregion, as well as the TNF1, TNF3, and TNF4 regions of the TNF-*α* gene in LLLT-treated cells (Figure 6(e)).

**Figure 7:**
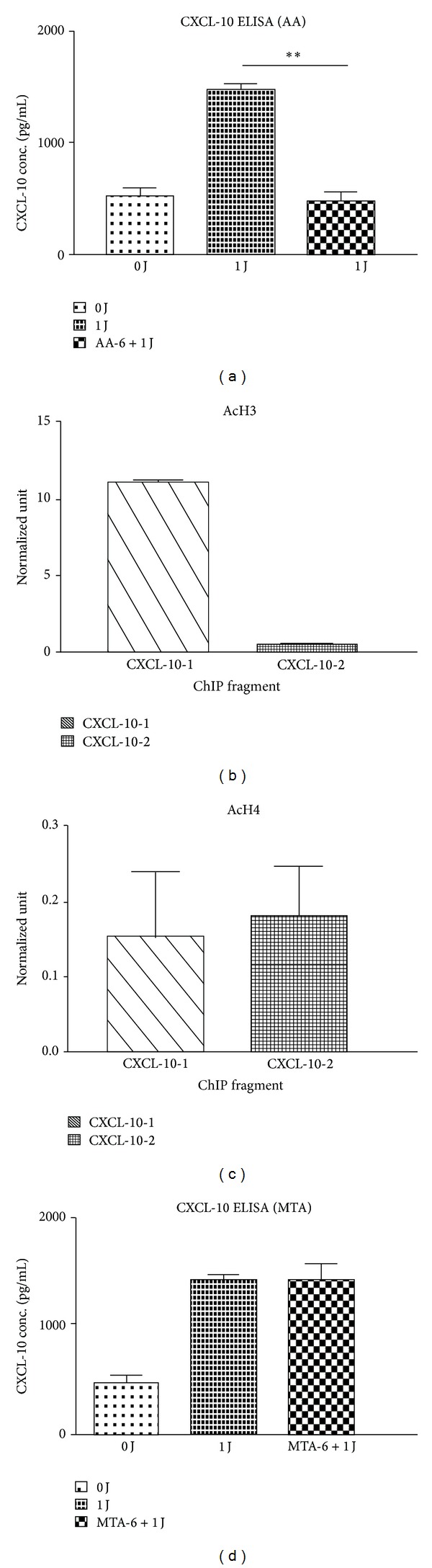
LLLT-induced IP-10 expression in human monocytes via inhibiting histone acetylation, but not trimethylation. The levels of IP-10 expression in LLLT-treated THP-1 cells in the presence or absence of AA (a) and MTA (d) using ELISA assay. ChIP analyses of the relative levels of (b) acetylated H3 (AcH3) and (c) acetylated H4 (AcH4) at the *IP-10* gene locus encompassing the following subregions relative to the transcription start site: CXCL10/IP-10-1: +9 to −172; CXCL10/IP-10-2: −444 to −622. The relative levels were normalized to the input DNAs and shown as mean (±SD) of 3 individual study subjects. **P < 0.01 was considered significant versus without the addition of inhibitor.

Next, we investigated whether LLLT-induced IP-10 expression in human monocytes was induced by increasing histone acetylation and trimethylation. Pretreatment with AA reversed LLLT-induced IP-10 expression in THP-1 cells (Figure 7(a)). The results correspond to the five experiments using THP-1 cells. ChIP analysis also showed increased levels of H3 at the proximal promoter subregion CXCL10-1 in the *IP-10* gene in LLLT-treated cells, whereas H4 occupation did not increase (Figures 7(b) and 7(c)). Pretreatment with MTA did not reverse LLLT-induced IP-10 expression in THP-1 cells (Figure 7(d)). Therefore, these findings suggested that the effect of LLLT on M1 polarization is associated with the cellular regulation of differential histone modification.

## 4. Discussion

Polarization of T cells and macrophages with cytokines influences every aspect of the immune response, including innate and adaptive immunity [1–3]. It is important to understand and be able to control macrophage polarization to eventually be able to enhance our immunity and treat immune disorders.

Over the last decades, we have witnessed an increasing prevalence of allergic diseases, which are relatively common and often debilitating diseases. This trend has posed a significant public health problem. Allergic diseases are caused by elevated Th2 cells, but the reason for this preferential activation is unclear. Macrophages are the major antigen-presenting cells involved in the induction of the primary immune response and play a critical role in immunity. IFN-r polarization, occurring through either classical or M1 activation, programs monocytes for phagocytosis, tumor resistance, and allergy suppression. It is also important to understand how to modulate the function of macrophages, induce M1 immunity to promote intracellular killing and tumor resistance, and prevent allergic reaction. LLLT is a form of light therapy with therapeutic effects on living tissues. In this study, 660 nm LLLT promoted M1 polarization and cytokine and chemokine mRNA and protein expression. Therefore, the effect of LLLT on monocyte polarization may be a potential treatment for allergic diseases and may also promote immunity to viral infections and tumors. The optimal dose of 1 J/cm^2^ may be more effective for promoting M1 immunity than 2 J/cm^2^ or 3 J/cm^2^.

TNF-*α* is an endotoxin-induced cytokine that causes necrosis and death of tumors and is also a pro-inflammatory cytokine predominantly released by macrophages [26]. Not only is TNF-*α* a pro-inflammatory cytokine, but it is also an immunoregulatory molecule that can modify the balance of T regulatory cells [27]. In addition, TNF-*α* is a central cytokine that triggers inflammation in rheumatoid arthritis (RA), indicating that the inhibition of TNF-*α* is an effective treatment strategy for RA [28]. CXCL10 plays an important role in resistance to and elimination of viral infections. Following a viral infection, CXCL10/IP-10 is secreted by bronchial epithelial cells, and Th1 cells are recruited via CXCR3 to eliminate the intracellular pathogen [29]. Baseline CXCL10 serum concentration is linked to the outcome of antiviral therapy in monoinfected hepatitis patients, as well as in patients coinfected with HIV [30, 31]. MCP-1/CCL2 is one of the key chemokines that regulate migration and infiltration of monocytes and macrophages. Both CCL2 and its receptor CCR2 have been shown to play vital roles in numerous diseases. The movement of monocytes from the blood stream across the vascular endothelium is required for both regular immunological surveillance and inflammatory response [32]. CCL2 inhibits the viral attachment of Human immunodeficiency virus (HIV-1) to the CCR2 and CCR5 coreceptors [33]. Additionally, the expression of all M1 polarization cytokines and chemokines is promoted by LLLT. Therefore, LLLT may be useful to promote antiviral immunity but may not be a suitable therapy for autoimmune or rheumatoid diseases.

Considering the importance of M1-polarized macrophages in various disease contexts, especially immunity to intracellular microorganisms and tumors, we examined the effects of different doses of LLLT on the expression of M1-polarized macrophages related cytokines and chemokines by using human THP-1 monocytes and provided evidence supporting the effects of LLLT on macrophage function. In this study, our results showed that after five days of muscular lesion, the activities of complex II and succinate dehydrogenase elevated considerably in contrast to the control group. Moreover, our results demonstrated that LLLT significantly increased the activities of complexes I, II, III, and IV and succinate dehydrogenase compared to the muscle injury group without treatment [24].

This study also shed light on the mechanisms of epigenetic regulation by LLLT in immune cells. Modifications on histones, such as acetylation or trimethylation at H3K4, H3K36, and H3K79, are associated with gene activation [20]. These modifications are usually carried out by a variety of histone acetyltransferases or methyltransferases [20]. Recently, histone modification has become a new target for antiallergy drug development [34]. In this study, LLLT induced histone H3 and H4 acetylation and H3K4 trimethylation in the TNF-*α* gene promoter area. LLLT also induced histones H3 acetylation in the *IP-10* gene promoter region but did not induce acetylation of histone H4. These results suggest that epigenetic regulation could be one of the important mechanisms by which LLLT modulates M1-related cytokine and chemokine expression.

In this study, 660 nm LLLT appeared to be a potent enhancer of the production of pro-inflammatory cytokines and M1-related chemokines in monocytes. M1-related immunoregulations play important roles in the antiviral and antitumor immunity and the pathogenesis of inflammation in autoimmune diseases. Because TNF-*α*, MCP-1, and IP-10 are important indicators of LLLT-induced M1 polarization, LLLT may promote anti-viral and anti-tumor immunity but enhance autoimmune and rheumatoid diseases. LLLT may be a potent immune-enhancing agent that is suitable for the treatment of allergic diseases but may not be a good therapy for autoimmune and rheumatoid disorders.

## Figures and Tables

**Table 1:** Laser characteristics.

Laser	Quantity					
	Total energy (J/cm^2^)	Power output (mW)	Spot area (cm^2^)	Time (sec)	Energy density (J/cm^2^)	Power density (mW/cm^2^)
660 nm	1.0	6	7.5	1250	1.0	0.8
	2.0	6	7.5	2500	2.0	0.8
	3.0	6	7.5	3750	3.0	0.8

808 nm	1.0	170	3.8	22.4	1.0	44.7
	2.0	170	3.8	44.8	2.0	44.7
	3.0	170	3.8	67.2	3.0	44.7
